# A refined diagnostic approach for interstitial lung disease: efficient and safe transbronchial cryobiopsy using a 1.1-mm cryoprobe

**DOI:** 10.3389/fmed.2025.1745802

**Published:** 2026-01-12

**Authors:** Qinghua Zhang, Jinyan Yu, Xiaohao Zhang, Tiangang Ma, Yan Wang

**Affiliations:** 1 Department of Respiratory and Critical Care Medicine, The Second Hospital of Jilin University, Changchun, Jilin, China; 2 Department of Cardiology, The Second Hospital of Jilin University, Changchun, Jilin, China

**Keywords:** Transbronchial lung cryobiopsy, 1.1-mm cryoprobe, interstitial lung disease, efficiency, safety

## Abstract

**Background:**

Transbronchial lung cryobiopsy (TBLC) has emerged as a less invasive alternative to surgical biopsy for diagnosing interstitial lung disease (ILD). However, data on the performance of thinner cryoprobes remains limited. This study aimed to evaluate the diagnostic utility, safety and pathological spectrum of TBLC using a 1.1-mm cryoprobe in a real-world cohort.

**Methods:**

We retrospectively reviewed 52 patients with ILD who underwent TBLC with a 1.1-mm cryoprobe from July 2023 to July 2025. Procedures were performed under digital subtraction angiography guidance using a standardized protocol. Primary outcomes were diagnostic yield and pathological spectrum; secondary outcomes included specimen characteristics and procedure-related complications.

**Results:**

The study demonstrated a high diagnostic yield of 88.5%. Multidisciplinary discussion diagnosis (MDD) revealed a diverse diagnostic spectrum: known-etiology ILD (50%) with hypersensitivity pneumonitis being the most common; idiopathic interstitial pneumonias (19.2%), mainly nonspecific interstitial pneumonia; and rare ILD (19.2%) with pulmonary alveolar proteinosis being the most common. The mean number of specimens obtained per operation was 3.5 ± 1.2, with a median specimen diameter of 5 mm (IQR: 4–6). The rate of severe bleeding was low at 3.8%, and there was no extremely severe bleeding. Pneumothorax occurred in only 1.9% of cases and managed without chest tube drainage.

**Conclusion:**

TBLC with the 1.1-mm cryoprobe is a highly informative and safe diagnostic method for ILD. It obtains high-quality samples that enable accurate classification, particularly in challenging cases, supporting its key role in minimally invasive diagnosis and precision in pathological subtyping.

## 1 Introduction

Interstitial lung disease (ILD) is a group of heterogeneous lung diseases that mainly involve the interstitium of the lungs and are mainly characterized by fibrosis and inflammatory cell infiltration as pathological changes. The histological changes of interstitial lung diseases are complex and diverse, and they are an extremely important part of the diagnosis of ILD. The means of obtaining histological specimens include surgical lung biopsy (SLB), transbronchial cryobiopsy (TBLC), transbronchial lung biopsy (TBLB), and percutaneous lung biopsy, etc. Although surgical lung biopsy can improve the accuracy of the diagnosis of interstitial lung diseases, it is highly invasive and has certain limitations. Although TBLC has a lower diagnostic yield compared to SLB, the latest TBLC guideline by European Respiratory Society recommends TBLC as the pathological diagnostic method for ILD due to its lower adverse event rates (1, 2). In addition, its better diagnostic efficacy than TBLB have been confirmed in many studies (3, 4). Therefore, in recent years, TBLC has been gradually applied in the pathological diagnosis of ILD. Obtaining tissue specimens through TBLC and conducting multidisciplinary discussions (MDD) enable the diagnosis of most ILD (1).

Traditionally, 2.4-mm, 1.9-mm and 1.7-mm diameter cryoprobe have been used for TBLC (5, 6). However, these non-ultrathin cryoprobes have limitations in sampling. The thickness and stiffness of their tips limit the access to certain areas, and they are not flexible enough, thus making the cryoprobes difficult to penetrate deep into the subpleural region and the apical segment of the upper lung lobe (6, 7). Against this backdrop, the 1.1 mm ultra-thin cryoprobe has been developed for TBLC (8). A recent study comparing 1.1-mm and 1.9-mm cryoprobes found that there was no difference between the two groups in specimen quality and diagnostic rate, and the risk of moderate bleeding during the use of the 1.1 mm cryoprobe was reduced (9). In addition, Kho and his colleagues demonstrated that the 1.1-mm ultrathin cryoprobe had a dramatic improvement over the 1.9-mm non-ultrathin cryoprobe when diagnosing lesions located in the apical segment of upper lobes and near the pleura (8). Our center has applied the 1.1-mm diameter cryoprobe for TBLC of ILD basing on the cryoprobe’s advantages. This study aimed to evaluate the diagnostic value, safety, and clinical impact of a 1.1-mm cryoprobe by retrospectively analyzing the clinical data of 52 ILD patients who underwent TBLC at our center.

## 2 Materials and methods

### 2.1 Study subjects

This retrospective cohort study included patients with suspected ILD who were approved for TBLC by the MDD and subsequently underwent the procedure between July 2023 and July 2025 in the Second Hospital of Jilin University. All the subjects signed the informed consent form. Inclusion criteria included the adult patients (age≥18 years) with clinically suspected ILD underwent the TBLC procedure using a 1.1-mm cryoprobe, and they had complete clinical, radiological, and pathological data available for multidisciplinary discussion (MDD). Exclusion criteria included undergoing anticoagulant therapy, currently using antiplatelet drugs, severe heart failure, severe pulmonary hypertension, uncontrolled hypertension or malignant arrhythmia, severe valvular heart disease or hypertrophic obstructive cardiomyopathy, severe respiratory failure, stroke in the past 3 months, uncontrolled epilepsy, severe liver and kidney dysfunction, allergy to anesthetic drugs, severe hematological diseases (including platelet count <50 × 10^9^/L), cachexia, cases with insufficient follow-up data to reach a final MDD diagnosis, etc. This study was approved by the ethics committee of The Second Hospital of Jilin University (approval number: 2025336).

### 2.2 Bronchoscopy procedures

**Figure 1:**
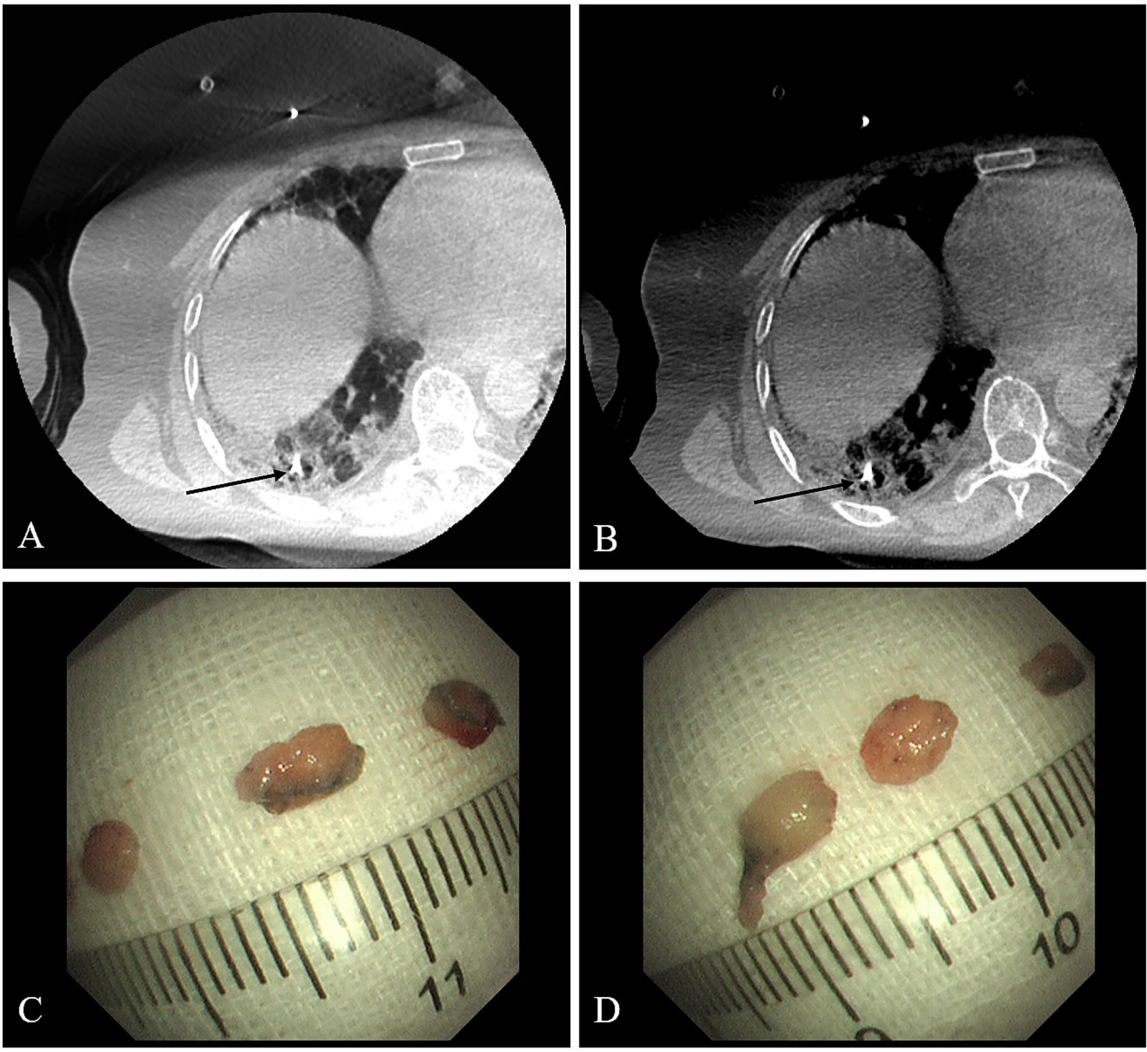
Digital subtraction angiography images during TBLC **(A,B)**. The cryoprobe tip (black arrow) is positioned approximately 0.5–1.0 cm from the pleural surface. Measuring the longest diameter of the cryobiopsy specimen using a ruler **(C,D)**.

Bronchoscopy procedures were performed under general anesthesia. A rigid bronchoscope (Karl Storz, Tuttlingen, Germany) was introduced into patient’s trachea under the guidance of the flexible bronchoscope BF-1TQ290 (Olympus, Japan). The target area of interstitial lesion was selected based on the chest CT imaging, and then the flexible bronchoscope P-290 (Olympus, Japan) or XP-290 (Olympus, Japan) was guided to the corresponding bronchi. The 1.1-mm cryoprobe was then inserted through the bronchoscope’s working channel, advanced until encountering the pleura and then retracted. Under the digital subtraction angiography (DSA, Azurion7M20, Philips, Netherlands), the cryoprobe was confirmed to reach the target area and adjusted so that the tip was 0.5–1.0 cm away from the pleura (Figures 1A,B). After freezing the for 3–9 s, the cryoprobe and the bronchoscope were withdrawn together (10). The frozen specimens were thawed on the sterile gauze in the room temperature, and then the bronchoscope was immediately inserted again and reached the target area, while checking whether there was severe bleeding. Meanwhile, the assistant transferred the tissue to fixative after measuring the diameter with a ruler (Figures 1C,D). Generally, 3–6 specimens were taken from each patient. CBCT would be taken after TBLC operation to check pneumothorax.

The degree of bleeding after TBLC was classified as follows: (1) Grade 0, no bleeding; (2) Grade 1, mild bleeding (for example, the bleeding can be cleared by negative pressure suction without other bronchoscopic measures); (3) Grade 2, moderate bleeding (for example, endoscopic interventional hemostasis, or local injection of ice saline, or 1:10000 ice epinephrine dilution); (4) Grade 3, severe bleeding (for example, bronchial occlusion balloon or surgical intervention is required, and using systemic anticoagulants); (5) Grade 4, extremely severe bleeding (for example, causing hemodynamic or respiratory instability, requiring blood transfusion, intubation, cardiopulmonary resuscitation, or admission to the intensive care unit).

### 2.3 Pathological analysis

The pathological diagnosis presented in the pathology report was made by at least two experienced pulmonary pathologists who independently review the slides. In case of discrepancies, a consensus should be reached through consultation between the two pathologists or by inviting a third pathologist for further evaluation.

### 2.4 Multidisciplinary discussion

The MDD team consisted of physicians from the department of respiratory medicine, department of radiology, and department of pathology. The final clinical diagnosis presented in the medical records was comprehensively derived through MDD based on high-resolution computed tomography (HRCT), pathology, clinical manifestations, and laboratory tests.

### 2.5 Statistical analysis

Statistical analyses were performed using SPSS (version 25.0). Continuous data are presented as mean ± standard deviation or median (interquartile range). Categorical data are expressed as frequency (percentage).

## 3 Results

### 3.1 Patient characteristics and baseline physiology

To reduce selection bias, we enrolled a consecutive series of all eligible patients within the specified time period. A total of 52 patients with ILD who underwent TBLC were ultimately included in the study. The baseline characteristics of the patients are shown in Table 1. The study population had a mean (± standard deviation) age of 55.9 ± 9.4 years and included 32 male (61.5%) and 20 women (38.5%). With regard to smoking history, 31patients (59.6%) were never-smokers, and 21(40.4%) were current smokers. Baseline pulmonary function data available for 39 patients are summarized. The main reasons for missing data included patient intolerance to the procedure and unretrievable external records. The retrievable data demonstrated a mild restrictive ventilatory pattern. Pulmonary function tests demonstrated a mild restrictive ventilatory defect, characterized by a reduction in total lung capacity (TLC: 72.7 ± 16.0%pred). This was accompanied by a mild decrease in forced vital capacity (FVC: 84.5 ± 16.9%pred). The absence of airflow obstruction was confirmed by a preserved FEV₁/FVC ratio (80.7 ± 10.7%) and a proportional reduction in FEV₁ (82.0 ± 16.0%pred). The most significant physiological impairment was a severe reduction in gas transfer, as evidenced by the diffusing capacity for carbon monoxide (DLCO: 52.2 ± 20.2%pred).

**Table 1:** Patients’ characteristics.

Characteristics	Values
Patients	52 (100)
Age years	55.8 ± 9.4
Sex	
Female	20 (38.5)
Male	32 (61.5)
Smoking status	
nonsmoker	31 (59.6)
smoker	21 (40.4)
Comorbid conditions	
Coronary heart disease	3 (5.8)
Hypertension	7 (13.5)
Connective tissue disease	3 (5.8)
Pre-existing lung disease	3 (5.8)
Pre-existing tumor	3 (5.8)
Cirrhosis	1 (1.9)
Exposure history	
Exposure to agents suspected to cause HP	2 (3.9)
Exposure to dust atmosphere	1 (1.9)
Lung function	
TLC % pred	72.7 ± 16.0
FVC % pred	84.5 ± 16.9
FEV1% pred	82.0 ± 16.0
FEV1/FVC	80.7 ± 10.7
DLCO % pred	52.2 ± 20.2

Data are present in *N* (%) or mean ± SD. HP, hypersensitivity pneumonitis.

### 3.2 Procedural details and safety outcomes

Data on TBLC procedures and the complications are shown in Table 2. Because the 1.1-mm cryoprobe is flexible enough to advance to bronchi of both upper lobes with an acute angle, 25% of the biopsies were performed in the right or left upper lobe, with the remainder obtained from the lower lobes (75%). A single pulmonary segment was sampled in 30 procedures (57.7%), while two segments were sampled in 22 procedures (42.3%). Freezing time data were unavailable for 3 of the 52 patients due to missing records. For 29 (59.2%) patients, the freezing time was maintained at 5–6 s, reaching a maximum of 9 s in one patient. A median of 3 (IQR: 3–4) tissue samples were obtained per patient. A total of 57 tissue samples from 13 patients were available for size measurement. The longest diameter of the samples averaged 4.8 ± 1.2 mm, with a median of 5 mm (interquartile range [IQR]: 4 to 5 mm). Regarding safety, the procedure was well-tolerated. Bleeding was the most common adverse event, with the majority classified as mild (Grade 1, *n* = 46, 88.5%) and managed conservatively. Moderate bleeding (Grade 2) occurred in 4 cases (7.7%), requiring topical ice-cold saline and/or 1:10000 epinephrine, and severe bleeding (Grade 3) requiring systemic anticoagulants was observed in 2 cases (3.8%). Pneumothorax occurred in only 1 patient and did not require chest tube drainage. Only 1 patient required post-procedural mechanical ventilation.

**Table 2:** Tblc procedures and the complications.

Parameters	Values
Lobar location	
Right upper lobe or left upper lobe	13 (25)
Other lobes	39 (75)
Number of lung segments	
One	30 (57.7)
Two	22 (42.3)
Freezing time for each biopsy	
3 s ~ 4 s	4 (8.2)
5 s ~ 6 s	29 (59.2)
7 s ~ 8 s	15 (30.6)
9 s	1 (2.0)
Number of samples	3.5 ± 1.2, 3 (IQR: 3–4)
Greatest length of each sample	4.8 ± 1.2 mm, 5 mm (IQR: 4–5 mm)
Bleeding	
Grade 0	0 (0)
Grade 1	46 (88.5)
Grade 2	4 (7.7)
Grade 3	2 (3.8)
Grade 4	0 (0)
Pneumothorax	1 (1.9)
Post-procedural mechanical ventilation	1 (1.9)

Data are present in N (%), mean ± SD or median (IQR).

### 3.3 Diagnostic yield and multidisciplinary diagnosis

**Figure 2:**
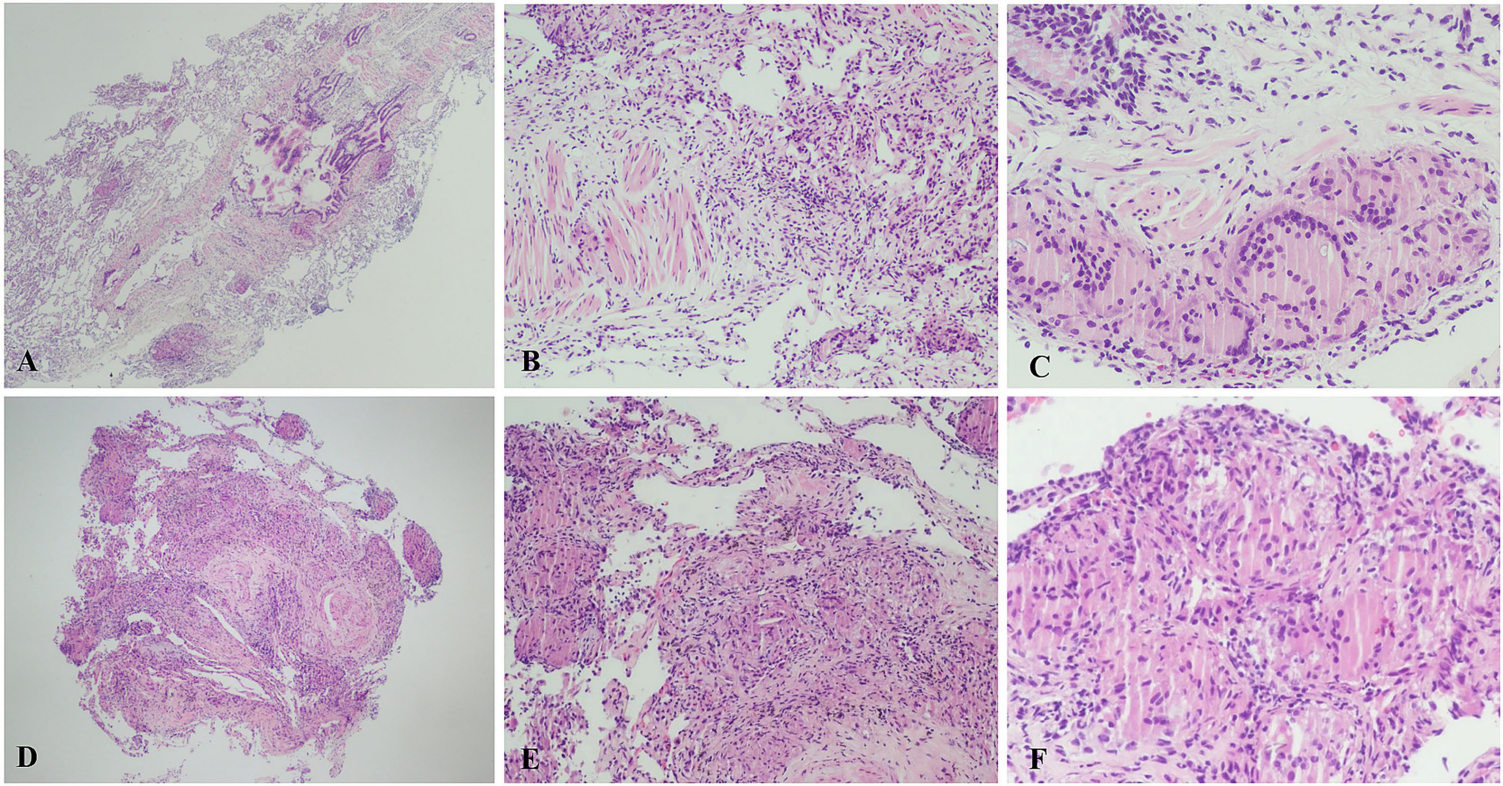
Histopathological features of HP **(A–C)** and pulmonary sarcoidosis **(D–F)** in TBLC specimens. **(A)** × 40 view showing a bronchiolocentric pattern of inflammation. **(B)** × 100 view showing the chronic inflammatory cells infiltration around surrounding small airways. **(C)** × 200 view revealing a poorly formed non-necrotizing granuloma. **(D)** × 40 view showing numerous non-necrotizing granulomas. **(E)** × 100 view showing compact granulomas with surrounding interstitial fibrosis. **(F)** × 200 view confirming a well-formed, dense non-necrotizing granuloma without necrosis.

The efficiency of TBLC on MDD was evaluated in all 52 patients. As summarized in Table 3, TBLC findings contributed to the final MDD diagnosis in 88.5% of cases (46/52). The classification of TBLC contributions (decisive/supportive) was based solely on their role in the integrative MDD diagnostic process and was independent of the final disease category. Accordingly, the contribution was decisive in 27 cases (51.9%), providing the pivotal evidence for a diagnosis that would not have been reached based on clinical and radiological data alone. In a further 19 cases (36.5%), the TBLC findings were supportive, adding confidence to a pre-existing diagnostic hypothesis. The procedure was non-diagnostic in 6cases (11.5%). Distribution of the final MDD-determined ILD diagnoses after TBLC is shown in Table 4. Diseases with a known etiology formed the largest category, encompassing 26 cases (50%). This group included exposure-related conditions such as HP (*n* = 8) and silicosis (*n* = 1). To illustrate the characteristic pathological findings, representative photomicrographs of a case with HP will be presented in Figures 2A–C, demonstrating features such as lymphocytic inflammation with poorly formed granulomas. Smoking-related diseases (RB-ILD and DIP, *n* = 4), and other secondary causes including infections and malignancies. Idiopathic interstitial pneumonias, representing diseases of unknown cause, accounted for 10 cases (19.2%). Idiopathic nonspecific interstitial pneumonia (NSIP, *n* = 8) was the most common subtype within this group. Notably, rare diseases constituted a significant proportion of our cohort with 10 cases (19.2%). This category was dominated by pulmonary alveolar proteinosis (PAP, *n* = 6), followed by sarcoidosis (*n* = 2). A representative histopathological image of sarcoidosis showing well-formed non-necrotizing granulomas will be provided in Figures 2D–F. Langerhans cell histiocytosis (*n* = 1), and diffuse panbronchiolitis (*n* = 1).

**Table 3:** Contribution of TBLC to the MDD diagnosis.

Level of contribution	Number of cases (%)
Decisive contribution	27 (51.9)
Supportive contribution	19 (36.5)
Non-diagnostic	6 (11.5)
Total	52 (100)

Data are present in *N* (%).

**Table 4:** Final ILD diagnosis based on MDD.

Category	Specific diagnosis	Number (%)
Known etiology	HP	8
	Connective tissue disease-associated ILD (CTD-ILD)	2
	Respiratory bronchiolitis-ILD (RB-ILD)	3
	Desquamative interstitial pneumonia (DIP)	1
	Silicosis	1
	Tuberculosis	3
	Secondary organizing pneumonia	2
	Post-infection pulmonary fibrosis	4
	Lung cancer	2
	Subtotal	26 (50)
Idiopathic	Idiopathic pulmonary fibrosis (IPF)	1
	Nonspecific interstitial pneumonia (NSIP)	8
	Combined pulmonary fibrosis and emphysema (CPFE)	1
	Subtotal	10 (19.2)
Rare diseases	Sarcoidosis	2
	Langerhans cell histiocytosis	1
	Diffuse panbronchiolitis	1
	Pulmonary alveolar proteinosis	6
	Subtotal	10 (19.2)
	Total diagnosed	46 (88.5)
Unclassifiable		6 (11.5)
Grand total		52 (100)

Data are present in *N* or *N* (%).

### 3.4 Analysis of non-diagnostic cases

Despite comprehensive evaluation, the diagnosis remained non-diagnostic in 6 cases (11.5%). A detailed review of these six cases confirmed that the sole reason for failure was the presence of non-specific fibrotic or inflammatory tissue only on pathological examination, which lacked the characteristic architectural features required for a definitive pattern diagnosis (e.g., UIP, NSIP, or granulomatous inflammation). Critically, there were no significant differences between these cases and the diagnostic cases in terms of specimen size or number of samples obtained. This indicates that the failure was not due to technical inadequacy in specimen procurement, but rather reflects the inherent diagnostic challenge of interpreting non-specific histological findings in the absence of a pathognomonic pattern.

## 4 Discussion

The accurate diagnosis of ILD necessitates a multidimensional integration of clinical, radiological, and pathological data within a MDD setting. TBLC has emerged as a minimally invasive technique that provides larger and more histologically preserved tissue samples compared to conventional forceps biopsy, thereby offering a promising alternative to surgical lung biopsy (SLB) for obtaining crucial pathological evidence. TBLC demonstrates a diagnostic yield exceeding 80% for most cases of ILD reported in the majority of previous large-scale studies and meta-analyses (11–13). In this study of 52 patients with diffuse interstitial lung disease, we demonstrated that the integration of TBLC findings into the MDD process allowed for a definitive final diagnosis in 88.5% of cases, providing substantial evidence to support the diagnostic utility of TBLC in real-world clinical practice.

The high diagnostic performance of TBLC was further underscored by its profound impact on refining initial diagnostic impressions. This was most strikingly exemplified in the diagnosis of HP in our study. Prior to TBLC, HP was suspected on clinical-radiological grounds in only 2 cases. However, following the integration of histopathological evidence from TBLC, the MDD established a definitive diagnosis of HP in 8 cases. This four-fold increase highlights a critical limitation of relying solely on non-invasive data, which can significantly underrepresent the true prevalence of HP due to its variable and often overlapping presentations. The pathological confirmation provided by TBLC was pivotal in identifying these additional cases, ensuring that patients received the correct diagnosis and subsequent management, which centers on antigen identification and avoidance, thereby directly altering clinical management pathways.

Our results are highly consistent with the recent prospective controlled trial by Hou et al. (9), which reported comparable diagnostic yields between 1.1 mm and 1.9 mm probes (80.4% vs. 79.8%) for ILD, affirming that thinner probes can achieve excellent diagnostic performance without compromising specimen quality. Furthermore, the high diagnostic performance of TBLC is echoed in studies beyond diffuse parenchymal lung diseases. Tanaka et al. (14) reported an outstanding diagnostic yield of 94% using a new 1.7-mm cryoprobe for peripheral pulmonary lesions. Although the patient population differs, the consistently exceptional yields from these studies powerfully highlight the technical success and robust diagnostic capability of modern TBLC techniques across various pulmonary pathologies.

The high diagnostic yield 88.5% in our study is underpinned by a standardized procedural approach that prioritized specimen quality. We obtained a mean of 3.5 ± 1.2 specimens per procedure with a median size of 5 mm, utilizing a freezing duration of 5–8 s in the majority of cases, parameters which align with best-practice recommendations (15) and directly support reliable pathological assessment.

Furthermore, the utilization of the 1.1-mm cryoprobe conferred a significant technical advantage in terms of accessibility. Its enhanced maneuverability allowed us to successfully target the upper lobes in 25% of all procedures, a site that is often under-sampled with larger, less flexible probes such as 1.9-mm and 2.4-mm cryoprobes. This technical advantage of the 1.1-mm cryoprobe likely contributed to the study’s robust diagnostic performance, particularly for diseases that predominantly affect the upper lobes, such as chronic hypersensitivity pneumonitis and pneumoconiosis. Our freezing protocol with 89.8% of procedures completed within 8 s achieving a high diagnostic yield demonstrated an effective balance between diagnostic efficacy and procedural safety.

The safety profile of TBLC utilizing the 1.1-mm cryoprobe in our study was favorable. The overall incidence of bleeding was acceptable, with the majority being mild. The relatively low rate of moderate bleeding (7.7%) aligns with the findings of Hou et al. (9). Importantly, the incidence of Grade 3 bleeding in our study (3.8%) was comparable to the 4.8% reported by Eom et al. (10) in their study on peripheral nodules using a similar 1.1-mm cryoprobe, reinforcing the safety advantage of 1.1-mm cryoprobes in mitigating severe bleeding risk.

The reported incidence of pneumothorax following TBLC varies considerably in the literature, ranging from 0% to as high as 31.7% (11, 16–20). This wide heterogeneity is largely attributable to differences in procedural techniques, operator experience, patient selection, and, crucially, the diameters of cryoprobes. The pneumothorax rate in our study was merely 1.9% (1/52), with no cases requiring chest tube drainage, positions our outcomes at the most favorable end of the reported spectrum. We attribute this exceptional safety outcome to a combination of factors: first, the utilization of a thinner 1.1-mm cryoprobe caused less parenchymal rip and air leakage; second, the stringent adherence to a standardized protocol under fluoroscopic guidance ensured a safe distance between the probe tip and the pleura; third, the procedure was performed by a highly experienced team in our center. In conclusion, when performed with 1.1-mm cryoprobe and meticulous technique, TBLC can achieve a diagnostically high yield with an exceptionally low risk of pneumothorax.

Notwithstanding these positive outcomes, a detailed analysis of the non-diagnostic cases provides crucial insight into a fundamental limitation of TBLC, and indeed of histopathology itself in ILD. In all six cases, the procedure successfully obtained adequate parenchymal tissue, however, the histology demonstrated only non-specific fibrosis or inflammation. This finding is clinically significant as it represents a “ceiling” for pathological diagnosis, where the tissue reaction is not distinctive enough to categorize into a specific disease entity. This scenario is often encountered in advanced fibrotic disease, where the original architectural pattern is obliterated, or in cases where the pathological differential remains broad despite adequate sampling. These cases underscore that a definitive diagnosis relies not only on obtaining tissue but on that tissue exhibiting a classifiable pattern. When TBLC yields such non-specific results, it necessitates even greater reliance on the MDD, where clinical and radiological context become paramount for assigning a probable diagnosis or guiding the decision for surgical biopsy.

This study characterizes the performance of the 1.1-mm cryoprobe within a well-controlled, single-center cohort, providing a benchmark for its diagnostic yield and safety when performed by specialized operators in ILD. These promising results not only support the procedural feasibility of the 1.1-mm cryoprobe, but also clearly define our objectives for the next phase of research, which will include multi-center trials to validate these findings and to assess their effect on long-term patient management.

## 5 Conclusion

In summary, this real-world study solidly validates the role of transbronchial lung cryobiopsy using a 1.1-mm cryoprobe as a cornerstone procedure in the diagnostic workup of interstitial lung disease. The technique achieves an optimal balance between high diagnostic yield and an exemplary safety profile, as evidenced by a remarkably low incidence of severe bleeding and pneumothorax. The procurement of high-quality, sizable tissue specimens was instrumental in revealing a diverse pathological spectrum through multidisciplinary discussion, thereby enabling precise diagnosis across common, rare, and challenging ILD cases.

Our findings specifically highlight that the 1.1-mm cryoprobe is not merely an alternative but a significant refinement in the TBLC technique. Its performance characteristics support its key role in advancing minimally invasive diagnosis and moving the field toward precision subtyping of ILD. Our future research would focus on multi-center trials to further quantify its advantages and standardize its application in clinical practice.

## Data Availability

The original contributions presented in the study are included in the article/supplementary material, further inquiries can be directed to the corresponding author.
